# Protective Activity of Aβ on Cell Cultures (PC12 and THP-1 after Differentiation) Preincubated with Lipopolysaccharide (LPS)

**DOI:** 10.1007/s12035-020-02204-w

**Published:** 2020-11-14

**Authors:** Benita Wiatrak, Katarzyna Balon

**Affiliations:** 1 Department of Pharmacology, Wroclaw Medical University, Mikulicza-Radeckiego 2, 50-345 Wrocław, Poland; 2 Department of Basic Medical Sciences, Wroclaw Medical University, Wrocław, Poland

**Keywords:** Amyloid-β, Lipopolysaccharide, Alzheimer’s disease, ROS, DNA damage

## Abstract

Amyloid-β (Aβ), the influence of which is considered the pathomechanism of Alzheimer’s disease, is also present in healthy people. The microbiome’s impact is also taken into account, where bacterial lipopolysaccharide (LPS) activates inflammatory processes and stimulates microglia via TLRs. Molecules of bacterial origin can co-create senile plaques with Aβ. This study evaluated the activity of physiological Aβ concentrations on neuronal and microglial cells after preincubation with LPS. Two cell lines were used in the study: PC12 cells differentiated with NGF and THP-1 cells differentiated with phorbol 12-myristate 13-acetate (PMA). Cells were incubated with LPS at concentrations of 1–100 μM for 24 h and then with Aβ_25–35_ at a concentration of 0.001 μM or 1.0 μM for another 24 h. The viability of the culture and free oxygen radicals and the number of DNA strand breaks in both cell lines were evaluated. Additionally, for PC12 cells, neural features were assessed. Stimulation of repair processes in the presence of Aβ was observed for both studied cell lines. There was a decrease in free radical level and DNA damage number compared to control cultures (cells treated with LPS and without Aβ). The neurotrophic activity of Aβ was observed—the effect on neurites’ growth even after the preincubation of PC12 cells with LPS. At the lowest concentration of LPS used, the increase in neurite length was about 50% greater than in the negative control. At low concentrations, Aβ has a protective effect on neuron-like PC12 cells pretreated with LPS.

## Introduction

Alzheimer’s disease (AD) is classified as a neurodegenerative disorder. This disease includes pathological loss of nerve cells and a reduction in the number of synaptic connections along with the loss of their plasticity. The disease develops asymptomatically for many years, and as a consequence, changes in the brain cause difficulties in daily activities, memory loss, mood swings, and changes in behavior. Over time, problems with recalling words or numbers and even disorientation in the family environment appear. The progress of science and technology influenced the development of medicine, the life span of people has significantly increased, and the risk of developing AD increases with age. Due to the growing number of patients, the financial outlay for medical care is growing and searching for possible effective therapies. There are still no effective drugs—the currently used drugs can only slow down the disease’s progression and do not reverse the changes that have already taken place. It is also a socially burdensome problem, and for family members.

The pathomechanism of AD is also ambiguous. In the literature, we can read many hypotheses about the development of the disease. The oldest of them is the cholinergic hypothesis. Currently used drugs contain active substances that inhibit the activity of acetylcholinesterase. The next two most popular are the tau protein hyperphosphorylation and the amyloid hypothesis—based on the toxicity of amyloid-β (Aβ) plaques. In addition to the toxic properties of Aβ, the positive features of this peptide are increasingly being reported. At the clinical/preclinical study stage, the negative effect of compounds with anti-amyloidogenic activity was demonstrated. The phenomenon of Aβ aggregation is a physiological response that shows a strong antimicrobial activity [1–3], by which it protects the brain against the effects of harmful molecules penetrating the blood‑brain barrier, by which it combines bacterial fragments and forms insoluble pods with them [2, 4]. Moreover, there is a relationship between the number of bacteria in the types most frequently represented among patients and AD biomarkers (including the level of Aβ_42_/Aβ_40_ in the cerebrospinal fluid) [5]. A small-group study in Japan showed that reduced numbers of Bacteroides and increased numbers of bacteria labeled “other” are better indicators of AD than traditional biomarkers such as ApoEε4 or a high result of VSRAD test (voxel-based specific regional analysis system for Alzheimer’s disease) [6]. Inflammation, more specifically neuroinflammation, is a factor that can link infections, changes in the microbiota, particles secreted and produced by microorganisms, and changes in the brain leading to the development of AD symptoms [7, 8]. Astrocytes, microglia, and pro-inflammatory cytokines have been observed in the vicinity of amyloid plaques and neurofibrillary tangles for a relatively long time—Alzheimer himself is the first to mention the inflammatory component of the disease image [7–9].

The post-mortem material of patients with AD revealed bacterial origin particles, including fragments of bacterial cells (peptidoglycan, flagellin, lipopolysaccharide—LPS) and bacterial amyloid [10]. The most important bacterial amyloid curli fimbriae are produced by *Escherichia coli* (they are biofilm components, biochemically similar to Aβ). It has been suggested that, due to the similar bacterial structure, amyloids can induce human proteins to adopt the pathological structure of the β-sheet [11]. LPS is a strong pro-inflammatory agent, and together with amyloid can co-create senile plaques [11–15]. At the same time, it causes cell damage and death in a dose-dependent manner. By interacting with the toll-like 4 receptor (TLR4), LPS activates the NF-κB, p38MAPK, or JNK signaling pathways, resulting in subsequent inflammatory cell damage. Therefore, this particular bacterial cell outer membrane compound is widely used to induce inflammation in neuronal cells in in vitro models of spinal cord injury (SCI) or various neurodegenerative diseases, including AD [16, 17]. It has been proved in many experiments that PC12 cells treated with LPS showed increased production of pro-inflammatory cytokines such as Il-1β, Il-6, and TNF-α, as confirmed by mRNA expression measurements [18–22]. It is generally recognized that the amount of microglia and amyloid is dependent on each other based on feedback. When the amount of Aβ aggregates increases, they can stimulate toll-like receptors (TLRs) and enhance the inflammatory response [23, 24]. After TLR2 and TLR4 activation, glial cells and astrocytes release pro-inflammatory cytokines (TNF-α, IL-1β, IL-6, IL-12, IL-18) and anti-inflammatory cytokines (IL-10 and TGF-β) [25, 26]. The presence of Aβ aggregates and damaged and dead cells in the brain tissue permanently stimulates proliferation and activates microglial cells, which maintains the stimulation of non-specific immune response, which affects the release of chronic inflammatory reaction mediators and intensification of the production of reactive oxygen and nitrogen species (ROS and RNS). The persistent stimulation of non-specific immune response is considered a key mechanism is driving the progression of neurodegeneration [24, 27, 28].

PC12 is a cancer cell line derived from the rat adrenal glands, which, after incubation with nerve growth factor (NGF), differentiates into cells that resemble biochemically and phenotypically sympathetic nerves [29]. The human THP-1 cell line is derived from a human with acute monocytic leukemia. THP-1 cell cultures incubated in the presence of phorbol 12-myristate 13-acetate (PMA) differentiate into microglia-like cells [30]. THP-1 cells treated with PMA are—in terms of morphology, expression of characteristic surface proteins, production of cytokines, and response to various stimuli—similar or even identical to human macrophages. Therefore, THP-1 cells present a suitable macrophage-like model, especially in response to TLR2 agonists (like LPS or amyloid-β) [31]. Expression of the M-phenotype activation markers, namely the pro-inflammatory cytokines Il-1β and TNF-α, corresponds to the expression observed in human monocyte-derived macrophage cultures [32–34]. THP-1 induced to differentiation with PMA, after contact with bacterial lipopolysaccharide, also secretes—among other eicosanoid derivatives—PGE2 (prostaglandin E2), which leads to suppression of M2-type polarization and enhanced expression of key markers of M1-phenotype (mainly Il-1β) [33]. The used concentration of 5 ng/ml and overall PMA treatment protocol in other studies proved to be sufficient to induce differentiation, ensuring CD14 expression and response to even weak pro-inflammatory stimuli [31, 35]. At the same time, the relatively low concentration of PMA used in this experiment ensured minimal cell death rate [30]. Both cell lines (PC12 and THP-1) are widely used as models in neurobiological research.

This study aimed to determine whether the presence of low doses of Aβ has a protective effect in cultures of neuron-like and microglia-like cells preincubated with LPS.

## Materials and Methods

### Cell Lines and Conditions

The study was carried out on two cell lines, often used as an in vitro model in neurobiological research. The first cell line was the PC12 line growing in suspension, derived from rat adrenal pheochromocytoma. The second cell line was the THP-1, human monocytic cells obtained from an acute monocytic leukemia patient. Both cell lines were purchased from ATCC. These cell lines were cultured with RPMI-1640. In the case of PC12 cells, the medium was supplemented with 10% donor horse serum (DHS; EuroClone, Italy), 5% fetal bovine serum (FBS; Biological Industries, USA) with 2 mME L-glutamine and 25 μg/ml gentamicin. In contrast, for THP-1 cells, the same L-glutamine and gentamicin concentrations and only 10% FBS were applied. These media were used for standard culture. Cell morphology and confluence were assessed using a microscope at least twice a week. The medium with cells was collected into previously prepared and described centrifuge tubes and centrifuged at 1000×*g* for 5 min. Then, the supernatant was removed, and fresh medium was added. THP-1 cells were pipetted and counted with a Bürcker chamber. However, the pellet of PC12 cells had to be broken with a syringe with a needle (twice each needle with a diameter of 12, 9, and 6) and then also counted in the Bürcker chamber. For the cell lines used to constitute an appropriate neurobiological model, it was necessary to differentiate them. For this purpose, 1 day after plating the cells, the primary medium was replaced with the differentiation medium. The differentiation medium for PC12 cells contained 100 ng/ml nerve growth factor (NGF) and for THP-1 cells 50 ng/ml phorbol 12-myristate 13-acetate (PMA). PC12 cells were differentiated for 3 days, and THP-1 cells for 5 days, with a medium replacement every 2 days. Cultures were incubated at 5% CO_2_, 95% humidity, and 37 °C. The tests used differentiation media supplemented similarly with L-glutamine and gentamicin, but the serum was reduced to 1% DHS for PC12 cells and 1% FBS for THP-1 cells.

To analyze the neural characteristics of PC12 cells, immunocytofluorescence was performed. For this purpose, the doublecortin (DCX) antibody was used. After incubation with NGF, cells were fixed with 100% cold methanol for 5 min. The cells were then washed three times with PBST (0.1% Tween 20 with PBS) for 5 min. Permeabilization was performed with 0.1% Triton X-100 in PBS for 10 min at RT. The blocking of non-specific antibodies was then carried out with a PBST solution supplemented with 1% bovine serum albumin (BSA) and 10% normal goat serum for 30 min. The antibody was prepared by diluting 1:500 in 1% BSA in PBST. The culture plates were washed three times with PBST for 5 min, and finally, the cells were treated with the antibody solution for 1 h at room temperature. The plates were washed again and observed under a fluorescence microscope (EVOS FL, Thermo Fisher Scientific).

### Tested Compounds

Lipopolysaccharide (LPS) (cat. no. L2630) from *Escherichia coli* and amyloid-β (25–35) (Aβ_25–35_) (cat. no. A4559) was purchased from Sigma-Aldrich, Saint Louis, USA. Both compounds were dissolved in distilled water to a final stock concentration of 1.0 mM. The stock solutions were stored at − 20 °C until use, but not longer than 6 months. For the biological experiments, the LPS was dissolved in the primary medium to a concentration range of 1–100 μM. The Aβ was also diluted at the primary medium but to concentrations of 0.001 μM and 1.0 μM.

### Modification of the Surface of Culture Plates

PC12 cells grow in suspension, so to be a neural model, they must be stimulated to adhere to a culture vessel surface. For this purpose, the surface of the culture plates was modified, and the wells were covered with a type I collagen solution (Sigma-Aldrich). The purchased collagen was dissolved in 0.1 M acetic acid to the concentration of 0.1% (w/v) and stored at − 20 °C until the end, but not longer than 6 months. To obtain a working collagen solution, the stock solution was diluted with distilled water to a final concentration of 0.01% (w/v). The volume of the working solution necessary to cover the entire surface was pipetted into the well. The plates were left at 4–8 °C overnight. The following day, the remaining solution was removed, and the covered plates were washed three times for 5 min with PBS. The prepared plates were stored at − 4 °C for no longer than a month. Plates were UV irritated for 30 min before use.

### Experiment Design

After the cells were differentiated, the medium was removed, and the LPS prepared in advance was added for 24 h at concentrations (1–100 μM). The next day the LPS solutions were removed, the culture was washed. The previously prepared Aβ was added at a concentration of 1.0 μM or 0.001 μM for the next 24 h in viability and genotoxicity assays or 1 h when the ROS level was measured. After this time, tests were performed to assess the viability of cell cultures, the level of free oxygen radicals, and DNA damage.

### Viability Assays

Viability for both cell lines was measured by the MTT assay. Besides, an additional test was performed for PC12 cells by measuring LDH (lactate dehydrogenase) release. These assays were performed at a cell density of 10,000 per well. After incubation of PC12 cells with Aβ, the supernatant was transferred to new culture plates. To carry out the LDH assay, the reaction mixture was added to the previously collected supernatants and left for 30 min in the dark. After this time, the stop solution was added, and the absorbance was measured at two lengths, 490 nm and 680 nm, using a Victor2 multi-plate reader. To perform the MTT assay, the cells of both lines were washed, and 1 mg/ml MTT (3-(4,5-dimethylthiazol-2-yl)-2,5-diphenyltetrazolium bromide) solution in MEM without phenol red was added into the culture. The cells were incubated for 2 h at 37 °C. The solution was then carefully removed, isopropanol was added, and plates were shaken for 30 min to dissolve the formazan crystals. Finally, the absorbance was measured at 555 nm using a Victor2.

### ROS Level

The ROS level was measured with DCF-DA assay. Cells were seeded at a density of 20,000 cells/well. After 1-h incubation with Aβ, the solution was removed and added a 20 μM DCF-DA (2′-7′-dichlorofluorescin diacetate) solution for the next 1 h at 37 °C. After removal of the supernatant, the cells were washed with PBS, and the fluorescence was measured at with excitation at 485 nm and emission at 535 nm using a Victor2.

### Fast Halo Assay

To assess the effect of Aβ on DNA damage in LPS-induced inflammation, the fast halo assay (FHA) was performed. The test was performed at a cell density of 50,000 per well. After incubation with Aβ, the supernatant was collected into pre-prepared tubes, and the culture was washed with PBS and harvested, and the TrypLE solution was added to the plates for 2 min at 37 °C. After collection of cell suspension, the tubes were centrifuged at 1000×*g* for 5 min. The supernatant was removed, and the pellet was resuspended in PBS and again centrifuged under the same conditions. The supernatant was removed again, and the pellet was resuspended to obtain a cell density of 30,000 in 30 μL DPBS and the tubes with cells were placed in a water bath at 37 °C. The cell suspension was then mixed with the agarose solution (1.25% low melting agarose in PBS), and the cell mixture was immediately pipetted between an agarose coated slide and a coverslip. This preparation was put on a cooling block and left to gel formation. The coverslip was removed, and preparation was put in lysis buffer overnight at 4 °C. The following day, the slides were moved in pH 13 buffer for 30 min at RT. After this time, the slides were transferred to a neutralization buffer twice for 5 min. Finally, the specimen was stained with 10 mM DAPI solution, and pictures were taken of 50 randomly encountered nuclei in each analyzed slide.

### Neuronal Features

To evaluate neuronal features, the length of the neurites was measured. Only cells whose neurite length was twice the body diameter were assessed. Additionally, the number of neurites for each cell was counted. Calculations were performed for 100 randomly selected cells (from numerous micrographs taken) in 5 independent experiments. The ImageJ image analysis software was used to count and measure neurites on microscopic images. Those neurites for which it was certain which the cell neurite originated and where it ends were assessed.

### Statistical Analysis

The results are presented as mean ± SEM. In this study, two control groups were used: negative control was cultured only primary medium without LPS, and Aβ and positive control were grown only with LPS without Aβ. The distribution of the results was normal, and the equality of variance was confirmed with Levene**’**s test, so statistical significance was calculated using parametric tests—ANOVA and the appropriate post hoc test. The *p* < 0.05 was considered the significance point. The Statistica v13.1 software was used for statistical analysis.

## Results

**Fig. 1:**
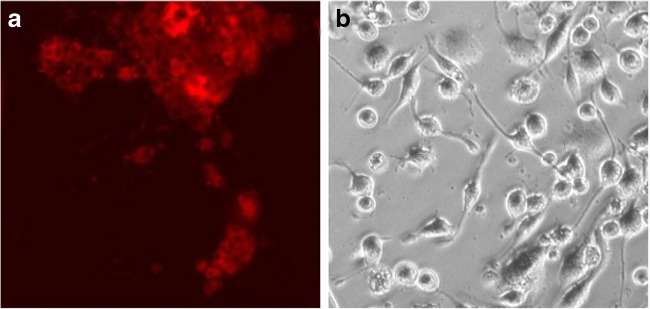
PC12 cells after immunocytofluorescence staining with doublecortin (DCX) antibody conjugated with PE fluorochrome (**a**); morphology of the THP-1 cells after PMA treatment (**b**)

Immunocytofluorescence staining was performed to check whether the cultures show neuron-like features after 72-h incubation of PC12 cells with NGF. For this purpose, PE-conjugated doublecortin (DCX) was used. The doublecortin protein is characteristic of young developing neurons [29, 36, 37]. After staining, it was shown that PC12 cells after 72 h of incubation with NGF-containing medium express doublecortin protein (Fig. 1a). Figure 1b shows the culture of THP-1 cells after incubation with PMA for 5 days. As a result of the incubation of the monocyte line in the medium containing PMA, the cell morphology changed—from cell suspension to cells adhering to culture vessels’ surface. Simultaneously, a change in the cells’ shape was observed—from spherical, almost identical cells, the culture became heterogeneous—flattened and round cells and cells with a significantly increased body size were observed along with the reorganization of the cytoskeleton and the development of branches, which constituted about 80% of cells in the field of view [38].

**Fig. 2:**
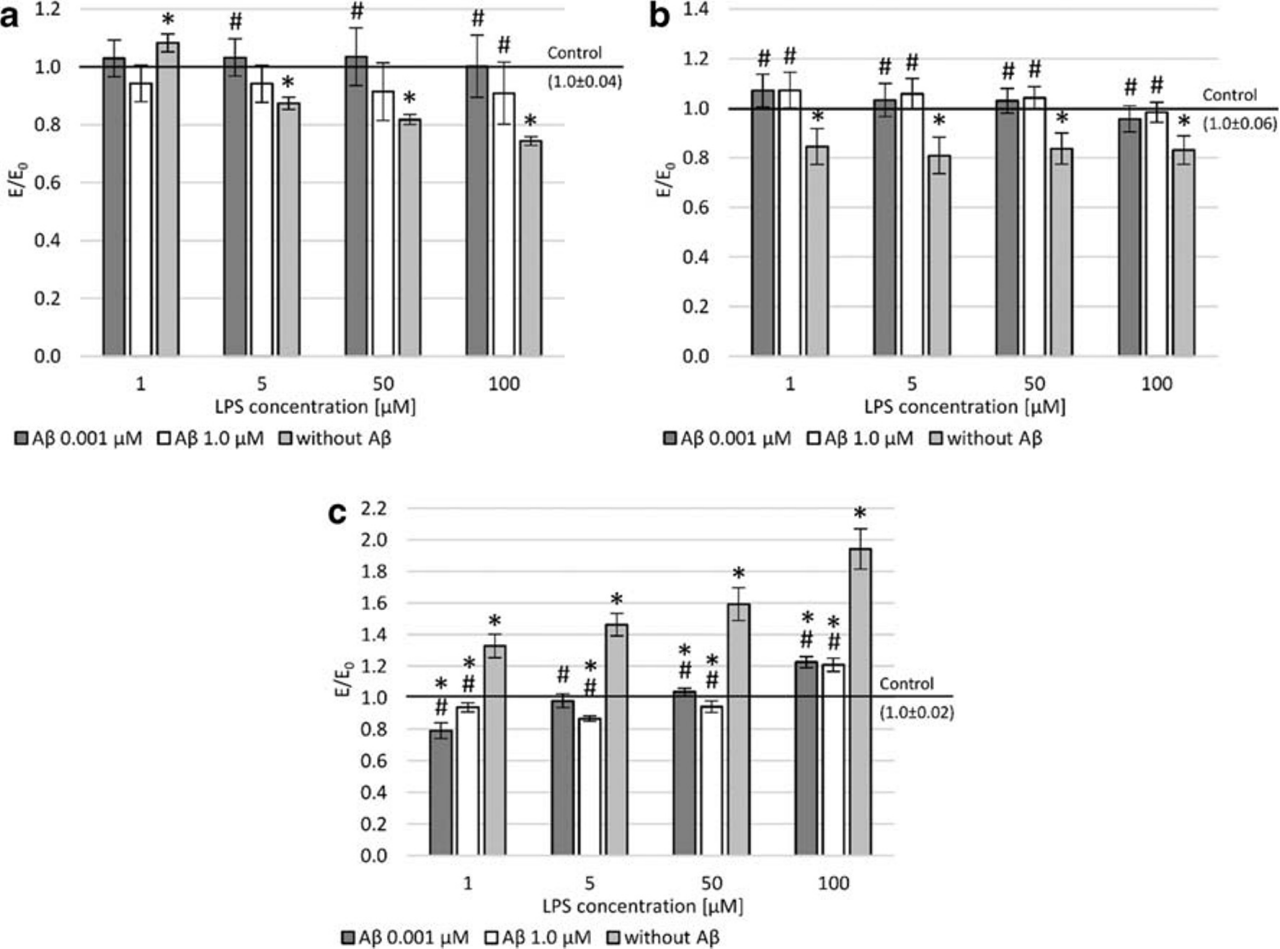
Effect of Aβ on PC12 cells (**a** and **c**) and THP-1 (**b**) cells preincubated with lipopolysaccharide (LPS); metabolic activity measured in MTT assay (**a** and **b**), cell viability measured in LDH assay (**c**); control—cell culture incubated without LPS and Aβ; * *p* < 0.05—significant difference compared to a negative control without LPS; # *p* < 0.05—significant difference compared to control preincubated with LPS

To investigate the effect of Aβ on the viability of two cell lines: PC12 after differentiation from nerve growth factor (NGF) and THP-1 after PMA treatment after prior LPS exposure, metabolic activity (MTT assay) was assessed (Fig. 2a and b). A statistically significant decrease in metabolic activity after incubation with LPS was observed in both PC12 and THP-1 cells (except 1 μM in PC12 cells). A concentration dependence was observed in the PC12 cell line. However, in the THP-1 line, regardless of the concentration used, a decrease in viability by about 20% was observed. Both 0.001 μM and 1.0 μM concentrations of Aβ influence the reduction of negative impact LPS on both cell lines.

The level of lactate dehydrogenase (LDH) release was also assessed, which indirectly indicates the number of necrotic cells in culture after incubation with LPS and then with Aβ. After PC12 cells were incubated in the presence of LPS, a concentration-dependent increase in the amount of released lactate dehydrogenase was observed. A statistically significant reduction in lactate dehydrogenase leakage was observed after incubation with Aβ. The regenerative effect of the 1.0 μM Aβ was significantly strong (reduction of LDH leakage was observed) even compared to the negative control (PC12 culture only on LPS and Aβ free medium) after preincubating the culture in the range 1–50 μM LPS. In contrast, a similar LDH leakage reduction effect (about 20%) compared to the negative control was seen with 0.001 Aβ only at 1.0 μM LPS.

**Fig. 3:**
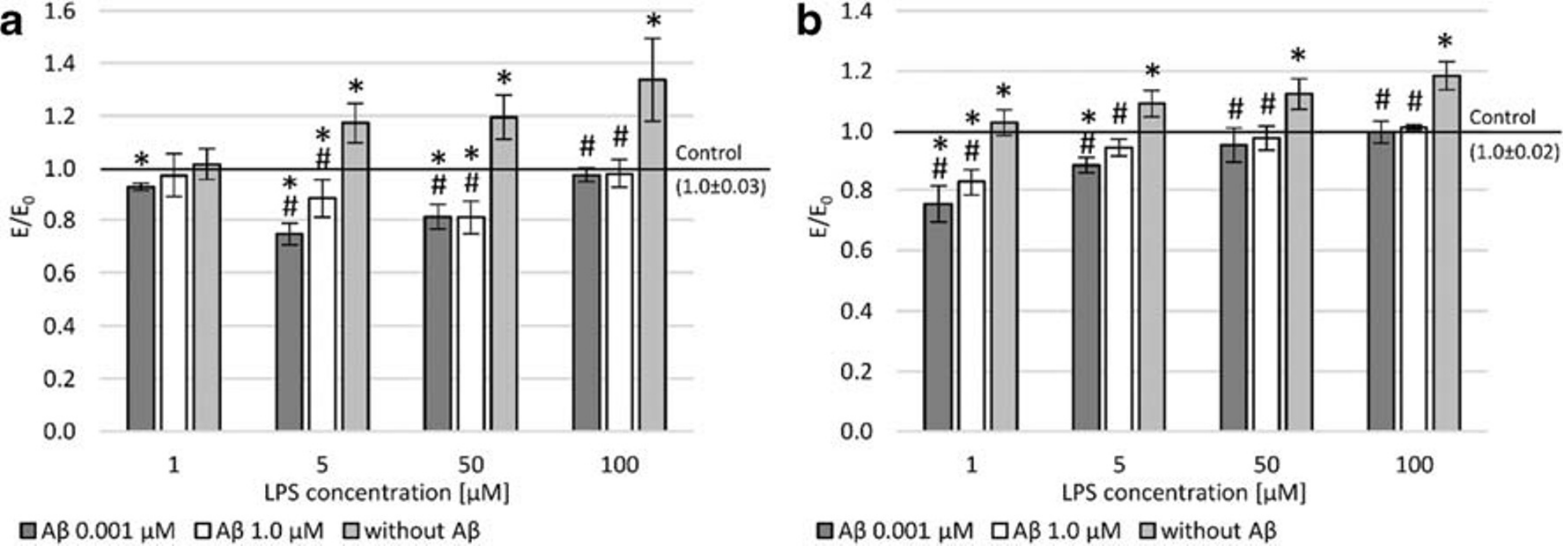
Effect of Aβ on PC12 cells (**a**) and THP-1 (**b**) cells preincubated with lipopolysaccharide (LPS); (**a** and **b**) DCF-DA assay; control—cell culture incubated without LPS and Aβ; * *p* < 0.05—significant difference compared to a negative control without LPS; # *p* < 0.05—significant difference compared to control preincubated with LPS

To assess intracellular ROS, accumulation was measured with the DCF-DA assay (Fig. 3a and b). This study demonstrated a higher level of reactive oxygen species in PC12 cells than in THP-1 cells after LPS treatment. In both cell lines, an increase in ROS dependent on LPS concentration was observed. After applying the concentration of 0.001 and 1.0 μM Aβ, the peptide’s regenerative effect was observed. That is, it reduced the level of free oxygen radicals (statistically significant). In both lines (PC12 and THP-1) after incubation with Aβ, regardless of the concentration used, a reduction in free oxygen radicals was observed to the level seen in the negative control after prior incubation with 100 μM LPS. A similar effect was demonstrated in the THP-1 line after incubation with a concentration of 50 μM LPS and PC12 cell line after treatment with a concentration of 1 μM LPS. In other cases, a statistically significant reduction in the level of free oxygen radicals was observed compared to the negative control.

**Fig. 4:**
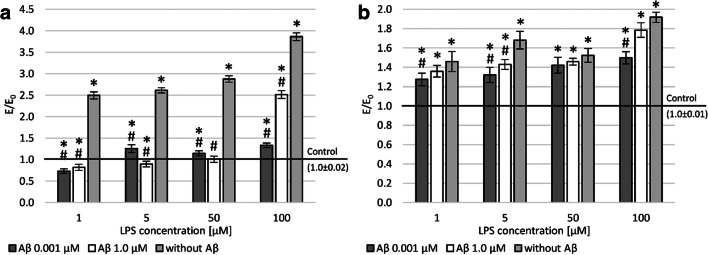
Effect of Aβ on PC12 cells (**a**) and THP-1 (**b**) cells preincubated with lipopolysaccharide (LPS); (**a** and **b**) fast halo assay; control—cell culture incubated without LPS and Aβ; * *p* < 0.05—significant difference compared to a negative control without LPS; # *p* < 0.05—significant difference compared to control preincubated with LPS

It is well known that high levels of ROS can lead to double-strand break (DSB). Accordingly, a fast halo assay (FHA) was performed, which measured the nuclear size of the halo (chromatin dispersion). In LPS-induced cells, the number of DSBs was almost twice and four times higher compared to the negative control at the highest concentration tested (100 μM) in the culture of THP-1 and PC12 cells, respectively (Fig. 4a and b). Aβ in concentrations of 0.001 and 1.0 showed a regenerative effect on damage to DNA strand breaks after prior incubation with LPS.

**Fig. 5:**
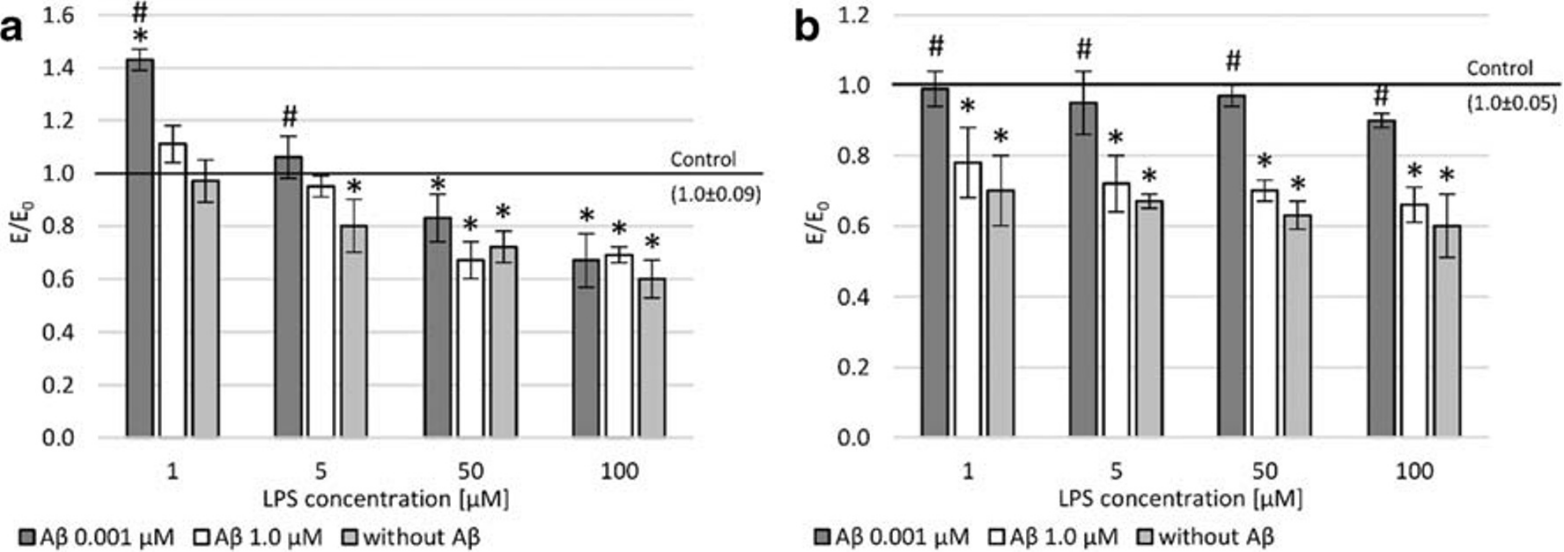
Effect of Aβ on PC12 cells preincubated with lipopolysaccharide (LPS); average length of neurites (**a**), an average number of neurites (**b**); control—cell culture incubated without LPS and Aβ; * *p* < 0.05—significant difference compared to a negative control without LPS; # *p* < 0.05—significant difference compared to control preincubated with LPS

To evaluate the impact on the neuronal feature of PC12 cells, the average length of neurites (Fig. 5a) and the average number of neurites (Fig. 5b) were analyzed. Incubation with LPS caused a statistically significant decrease in neurite length (to up 60% in higher concentration). For all concentrations of Aβ, an increase in the average length of measured neurites was shown compared to LPS-induced cells. The neurite growth was statistically significantly greater after applying Aβ at a concentration of 0.001 μM than at 1.0 μM. At the same time, this increase was comparable to the negative control.

**Fig. 6:**
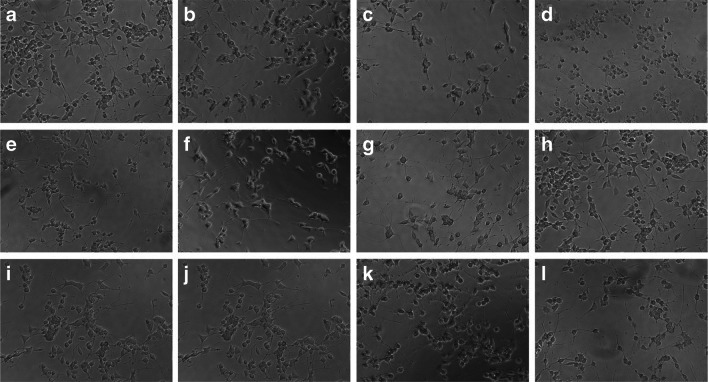
Effect of Aβ on PC12 cells preincubated with lipopolysaccharide (LPS); incubation without Aβ (**a**‑**d**), with Aβ at a concentration of 0.001 μM (**e**‑**h**), and with Aβ at a concentration of 1 μM (**i**‑**l**); LPS at a concentration of 1.0 μM (**a**, **e**, **i**), 5.0 μM (**b**, **f**, **j**), 50.0 μM (**c**, **g**, **k**), and 100.0 μM (**d**, **h**, **l**); objective magnification: × 10

The average number of neurites after incubation of cells with LPS was statistically significantly lower in the concentration range of 5–100 μM compared to the negative control. Moreover, the reduction in the number of neurites was concentration-dependent. An increase in the mean number of neurites was demonstrated after incubating the culture with Aβ at both 0.001 and 1.0 μM concentrations. The average neurite length was 40 μm for negative control. It is worth noting that after incubation with Aβ at a concentration of 0.001 μM, a neurotrophic activity could be observed, and the average length of neurites was about 58 μm (Fig. 5a). Figure 6 shows exemplary micrographs showing the morphology of PC12 cells. The longest neurites are in Fig. 6e (1.0 μM LPS and 0.001 μM Aβ), and the culture shown in Fig. 6d (100.0 μM LPS without Aβ) is characterized by the shortest neurites.

## Discussion

A growing body of evidence suggests that amyloid-β—considered for a long time to be a hallmark for pathology with no physiological function—may have antimicrobial properties as a part of the innate immune system. Aβ molecules share some striking similarities with recently recognized antimicrobial peptides (AMPs)—potent antibiotics with immunomodulatory properties produced in human bodies—together with its most thoroughly studied representative LL-37 protein. The tendency to self-assemble to form oligomers in pathology may lead to creating senile plaques typical for Alzheimer’s disease. Still, it has been proven that Aβ oligomerization enables binding, agglutination, and entrapment of certain pathogens while inhibiting their adhesion preventing the spread of infection and facilitating phagocytosis [1, 2, 39–41]. Aβ molecular structure, just like LL-37, contains a heparin-binding motif enabling attachment to carbohydrates being a part of the microbial cell wall [2, 42]. As studies show, Aβ can stop microbes from spreading and may kill viruses, fungi, and bacteria. Microbicidal properties of known AMPs usually stem from their capability to self-aggregate in the form of fibrils and interacting with negatively charged microbial plasma membranes (and sometimes mitochondria in line with the endosymbiotic theory of their origin), leading to uncontrolled ion leakage and death of attacked cell [4, 42, 43].

Numerous studies report that circulating concentrations of amyloid-β (including neurotoxic oligomers) vary immensely between AD patients [44], probably due to various measuring methods and inconsistency in the preanalytical treatment of specimens. It is already established that plasma levels of amyloid-β correlate poorly with patients’ clinical conditions [45]. Therefore in our study, we chose concentrations of soluble amyloid-β observed in brain homogenates and CSF (cerebrospinal fluid) of AD subjects that are ranging from nanomoles [46, 47] to micromoles [47–50]. Concentrations of 1 nM and 1 μM, respectively, were selected due to the neurotrophic properties of nanomolar concentrations of Aβ_25–35_ [51] and reported (dose-dependent) neurotoxicity of micromolar concentrations [52, 53].

As early as the 1990s, scientists tried to draw attention to the positive effects of Aβ in cell cultures. Luo et al. proved that in the culture of PC12 cells, Aβ at a concentration of 1 nM had a pro-proliferative effect (even stronger than BSA administration). Moreover, Aβ_25–35_ has been shown to have a stronger pro-proliferative activity compared to the two more frequently studied Aβ fragments, 1–40 and 1–42 [54]. The next step in demonstrating the positive effect of Aβ on cell cultures was to evaluate its effect on PC12 cells during differentiation. Yanker et al. observed that during the first 48 h of differentiation, the presence of Aβ_1–40_ showed a neurotrophic activity, while in the following days, it was neurotoxic. At the same time, Aβ_25–35_ was shown to have a stronger neurotrophic effect compared to other fragments, and the impact on the viability was not so pronounced [51]. The neurotrophic effect of Aβ_1–42_ (even at a concentration of 1.0 μM) was also demonstrated in the cultures of neural stem cells [55]. An interesting observation was also made by Arevalo et al., who conducted in vitro studies on neurons isolated from the mouse hippocampus. They added to the cell culture Aβ at a concentration of 20 nM and 800 nM. They found that the low concentration had a positive effect on the length of neurites but reduced the density. The opposite effect was observed at a higher concentration (800 nM) [56]. In our study, it was shown that amyloid in low concentrations has a regenerative effect on neuron-like cells (PC12) and influenced the viability and growth as well as the number of neurites in the culture after prior incubation of the culture with LPS. It has also been shown that physiologically low concentrations of Aβ have neurotrophic properties. We noticed that a low concentration of Aβ (0.001 μM) had a strong neurotrophic effect on neurite length, while the effect on neurite density was comparable to the control (without LPS).

Reactive oxygen species production catalyzed by bound metal (zinc or copper) and activating complement are also mediated by oligomers formation [39]. As a member of the AMP group, Aβ is an agent of the innate immune response in the immune-privileged brain, and its expression is heightened not only in AD but also in neuroborreliosis, neurosyphilis, HIV-related dementia, herpes simplex encephalitis, and *Chlamydia* brain infection [1, 40, 41, 57]. Further proving Aβ peptide’s engagement in immune response in the brain, apolipoprotein E is suggested to be a part of innate immunity and its homozygous variant ε4—the major genetic risk factor for sporadic Alzheimer’s disease—is associated with increased susceptibility to some pathogenic microbes including neurotropic viruses [39, 41]. Additionally, the brains of patients with AD tend to show higher viral or bacterial loads than their age-matched controls. A large proportion of Aβ-formed plaques contains particles and genetic material of viruses and bacteria [41]. We proved that fragments of bacterial cells, in this case, LPS, caused an increase in the level of free oxygen radicals after 24-h incubation of PC12 cultures with LPS. In contrast, the incubation of PC12 cell cultures with Aβ caused the scavenging of free radicals. At the same time, along with a decrease in the level of free oxygen radicals, the regeneration of DNA strand damage was observed.

Antimicrobial properties of Aβ, either synthetically produced or obtained from the brain and temporal lobe homogenates of AD patients and Aβ-overexpressing transgenic animals, have been proven in vitro for a variety of pathogenic microbes, including Gram-positive and Gram-negative bacteria, viruses like influenza A and herpes simplex virus type 1, and also *Candida albicans* [1, 4, 39, 41]. Aβ_1–42_ showed higher than Aβ_1–40_ antimicrobial activity and the ability to agglutinate pathogens, which might be connected to hydrophobicity and readiness to self-aggregate Aβ_1–42_. Observed effects were obliterated after treatment with anti-amyloid beta antibodies [1, 2, 40, 43]. In vivo experiments revealed a higher survival rate of Aβ-overexpressing 5xFAD mice infected intracerebrally with *Salmonella* Typhimurium with lower cerebral bacterial loads than their non-transgenic littermates [2, 39, 41]. Similarly, transgenic nematodes *Caenorhabditis elegans* producing Aβ_1–42_ isoform (GMC101) showed reduced mortality after *C. albicans* infection than control CL2122 worms [2, 39, 41].

Treatments targeted at amyloid-β oligomers and fibrils perceived then as purely pathological structures showed numerous side effects in clinical trials, including a higher risk of meningitis and upper respiratory tract infections. They increased blood‑brain barrier permeability leading to edema, micro-hemorrhages, and neurovascular disturbances [1, 39, 41, 42]. Adverse effects of anti-Aβ therapy seem to be directly or indirectly related to disturbed physiological functions of said peptide.

**Fig. 7:**
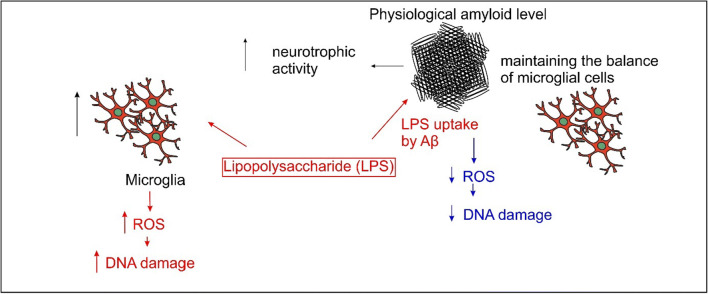
Likely mechanism of action of Aβ at physiological concentrations

Aβ senile plaques, which may also contain other proteins and fragments of bacterial cells, including LPS, cannot be effectively removed by microglial cells. These deposits strongly activate pro-inflammatory signaling pathways in brain tissue cells (including cytokines) and increase the production of reactive oxygen and nitrogen species [25]. The role of the activation of the non-specific immune response pathway in the development of neurodegeneration may be an attractive target for future therapy because post-mortem studies of brain tissue in people with mild cognitive impairment (MCI) showed a significant increase in the number of activated microglial cells (immunophenotype M2) and a significant increase in the activity of the NLRP3-caspase complex 1 [23]. An important mechanism driving the progress of neurodegenerative processes is the persistent inflammation caused by the activation of a non-specific immune response leading to the release of pro-inflammatory cytokines and the maintenance of neuronal oxidative stress. The presence of Aβ oligomers inside cells, extracellular deposits of Aβ aggregates in senile plaques, and the presence of damaged and dead cells in brain tissue can induce a non-specific immune response in Alzheimer’s disease. The neuroprotective task of astrocytes and microglia is the removal of apoptotic and necrotic cells and amyloid aggregates from the brain tissue [58, 59]. However, excessive proliferation of glial cells and their activation can exacerbate the inflammatory process, damage neurons, and affect the loss of synaptic spines, thereby reducing synaptic function [58–60]. At the same time, the multiplication and activation of microglia cells are associated with the constant presence of Aβ aggregates and damaged and dead cells in the brain tissue, which affects the increased production of ROS and reactive forms of nitrogen (RNS) and the release of chronic inflammatory mediators. Long-term stimulation of non-specific immune response is believed to be a key mechanism driving neurodegeneration progression [24, 27, 28]. In connection with the “vicious circle” between Aβ level and microglia amount, we wanted to see how bacterial particle (LPS) fragments affect microglia cells and then whether the physiological Aβ concentration would have a regenerative effect on LPS damage. The study showed that LPS increased the level of free oxygen radicals and damaged DNA strands in microglia cells. At the same time, incubation with Aβ at physiological concentrations caused less damage to both the DNA strand and the ROS level than after incubation with LPS. The incubation of microglia with LPS caused a decrease in cells’ metabolic activity, while incubation with Aβ resulted in activity similar to the negative control. This may indicate a persistent balance between the Aβ and the microglia. The presence of amyloid reduced the level of ROS, similar to the negative control. Only the regeneration of DNA damage was weaker. The likely mechanism of action of Aβ on nerve cells and microglia is shown in Fig. 7.

The condition of the patient’s microbiota may be of great importance in Alzheimer’s disease. The variety of microorganisms plays a significant role in digestion and providing the host with nutrients. However, if these organisms, their fragments, or substances produced by them cross the intestinal epithelium’s protective barrier, it can pose a serious threat. Microorganisms do not even have to leave the gastrointestinal tract to influence metabolism, immune response, and even host behavior, which may be related to the “microbiota-gut-brain axis”. The intestinal microbiota contacts the central nervous system through a complex network of signals consisting of neurotransmitters, conduction of stimuli through the vagus nerve and the autonomic nervous system, and secreted short-chain fatty acids (SCFAs), micro RNA (miRNA), small non-coding (sncRNA), and other active molecules, as well as changes in the permeability of the blood‑brain barrier (BBB) [14, 61–63]. Our study demonstrated the toxic effect of bacterial LPS on neuron-like and microglia-like cells, as well as the regenerative effect of low doses of Aβ. This is another step that challenges the amyloid hypothesis. It seems likely that high Aβ levels are only the brain’s response to an excess of harmful factors (including LPS) that lead to neuroinflammation.

This work’s limitation is the lack of analysis of the influence of co-cultures of neuronal cells and microglia. The authors plan to conduct such research in the next stage of work. It is also planned to determine the level of pro-inflammatory cytokines in cultures. Moreover, the concept of this study was based on the hypothesis of amyloid cascade and neuroinflammation as the main pathomechanism involved in Alzheimer’s disease and especially on the idea that amyloid-β, as a member of AMPs (antimicrobial proteins), could have a beneficial effect on neuronal cells in the presence of bacterial LPS and that disturbance in the physiological pathways involving Aβ and causing neuroinflammation might be the underlying cause of Alzheimer’s disease development [64–66]. However, emerging new studies suggest that a model based on tau protein pathology should also be considered in terms of the development and propagation of the disease. Tau burden correlates with clinical symptoms and is needed in some in vitro studies or animal models of AD for the appearance of neuronal damage [64, 65, 67, 68]. Therefore, the inclusion of tau protein in future research, in addition to amyloid-β, could result in a new and better understanding of the mechanisms leading to AD pathology, especially as there are still few experiments considering tau hyperphosphorylation as more than just an Aβ-induced phenomenon [68–71].

In conclusion, LPS in the entire range of concentrations tested increased ROS levels and frequency of DNA damage in THP-1 and PC12 cultures and did not affect neurite growth in PC12 cells. In contrast, Aβ at low concentrations [0,001–0.1 μM] did not increase toxicity, DNA damage, and ROS levels in THP-1 and PC12 cultures, while significantly increased neurite growth in PC12 cultures. Further research is needed to confirm the neuroprotective effect of these low (close to physiological) Aβ concentrations.

## Data Availability

The datasets generated and analyzed during the current study are available from the corresponding author upon reasonable request.
